# GenAI-enabled accounting information systems competence: Antecedents, perceived usefulness, AI ethics awareness, and career readiness outcomes

**DOI:** 10.1371/journal.pone.0362651

**Published:** 2026-11-13

**Authors:** Trung Kim Quoc Nguyen, Khanh Phuc Nguyen

**Affiliations:** 1 Faculty of Accounting and Auditing, Ho Chi Minh City Open University, Ho Chi Minh City, Vietnam; 2 Faculty of International Commerce, School of Business, Van Hien University, Ho Chi Minh City, Vietnam; Liverpool John Moores University, UNITED KINGDOM OF GREAT BRITAIN AND NORTHERN IRELAND

## Abstract

This study examines GenAI-enabled accounting information systems competence (GAISC) among accounting and auditing students and its relationships with GenAI readiness (GAR), perceived ease of use (PEOU), digital learning self-efficacy (DLSE), facilitating conditions (FC), perceived usefulness (PU), AI ethics awareness (AIEA), and career readiness (CR). The model is anchored in Socio-Technical Systems Theory (STS) and Social Cognitive Career Theory (SCCT), with technology readiness, TAM, self-efficacy, and facilitating-condition logic used as construct-level underpinning mechanisms. Survey data from 471 students at five Vietnamese universities were analyzed using PLS-SEM, complemented by a common latent factor diagnostic and Gaussian copula endogeneity assessment. The baseline PLS-SEM model showed statistically significant positive associations for all nine hypothesized relationships and explained 53.1% of the variance in GAISC, 45.6% in AIEA, and 52.9% in CR. Substantively, PU was the strongest baseline antecedent of GAISC (β = .427, f^2^ = .244), while GAISC showed a particularly strong association with AIEA (β = .675, f^2^ = .838). Endogeneity adjustment indicated that seven relationships remained robust; however, the relationships between FC and GAISC and between AIEA and CR became non-significant. Accordingly, these two paths are interpreted as baseline-supported but not robust after adjustment. The findings move beyond generic GenAI acceptance by positioning GAISC as an accounting-specific competence construct linked to ethical awareness and career readiness. Because GAISC and CR were measured through student self-reports and the evidence is cross-sectional, the results are interpreted as association-based rather than as demonstrations of objectively verified competence, employability, or causal effects.

## 1. Introduction and research gap

Generative artificial intelligence (GenAI) is reshaping accounting education by changing how students obtain explanations, generate analyses, and interact with accounting information. Recent evidence shows that GenAI can support help-seeking, feedback, engagement, and task completion, but also raises concerns about overreliance, superficial learning, and weakened critical evaluation [1–3]. These tensions are particularly consequential in accounting because professional work requires evidence, documentation, internal-control awareness, source reliability, and accountable judgment rather than plausible text generation alone. This broader digital transformation of accounting has also been documented across data mining, enterprise analytics, business intelligence, and contemporary AI research [4–7].

Accounting education therefore needs to move beyond the question of whether students accept or use GenAI. Contemporary pedagogical work emphasizes verification, reperformance, critical error review, and professional skepticism when students work with AI-generated accounting outputs [8]. More broadly, emerging AI-literacy research treats competent use as multidimensional, involving knowledge, use, evaluation, problem solving, and ethics rather than simple technological familiarity [9,10]. This distinction is central to the present study because its focal construct is GenAI-enabled AIS competence rather than technology-adoption intention. In the present survey, GAISC is measured through students’ self-assessment and is therefore not interpreted as objectively demonstrated task performance.

The distinction is also empirically relevant in Vietnam. Recent research on Vietnamese accounting students shows that technology readiness, perceived usefulness, perceived ease of use, AI literacy, social influence, and facilitating conditions are associated with AI adoption [11]. Adoption, however, does not establish that students perceive themselves as able to process accounting information with GenAI, verify outputs, detect inconsistencies, or exercise professional judgment in AIS-related tasks. The present study addresses this competence-oriented gap and further examines whether GAISC is associated with AI ethics awareness and career readiness, while assessing the robustness of the structural relationships to potential endogeneity.

The paper proceeds as follows. Section 2 reviews literature, establishes the STS and SCCT foundations, explains the construct-level underpinning mechanisms, and develops the hypotheses. Section 3 describes research design, sampling and consent procedures, measurement, common-method-bias diagnostic, and PLS-SEM/endogeneity strategy. Section 4 reports the results. Section 5 discusses the four most substantively important findings, Section 6 presents theoretical and practical implications, Section 7 outlines limitations and future research, and Section 8 concludes.

### Research gaps

Two interrelated gaps motivate this study. First, much of the emerging literature on AI among accounting students remains organized around adoption, usefulness, ease of use, readiness, or general AI literacy. These perspectives are important, but they do not establish whether students perceive themselves as capable of performing accounting-specific information tasks with GenAI. In AIS settings, competence requires more than access or willingness to use a tool; it involves processing accounting information, evaluating AI-generated outputs, detecting inconsistencies, considering controls, documenting evidence, and applying professional judgment [8,12,13]. The conceptual gap is therefore a shift from generic acceptance or literacy toward accounting-specific GAISC. Earlier AIS research likewise shows that accounting knowledge structures, representation formats, conceptual-model evaluation, and cognitive fit can shape information retrieval and evaluation performance [14–17].

Second, the substantive interpretation of survey-based structural relationships requires greater caution. Constructs such as usefulness, perceived competence, ethical awareness, and career readiness may be jointly influenced by unobserved learner or institutional characteristics, while facilitating conditions may partly reflect lecturer capability, curriculum quality, institutional digital maturity, or prior AI exposure. Conventional PLS-SEM significance alone does not resolve these concerns [18–20]. The study therefore combines baseline PLS-SEM estimates with Gaussian copula diagnostics so that statistically significant baseline relationships can be distinguished from relationships that remain robust after endogeneity adjustment.

### Contributions of the study

The study’s core contribution is to move the analysis of GenAI in accounting education beyond generic acceptance or use toward accounting-specific GAISC. Rather than treating usefulness, ease of use, readiness, self-efficacy, and support as parallel predictors of adoption, the model asks whether these learner, technology-appraisal, and contextual conditions are associated with students’ self-reported competence to process, verify, question, and apply GenAI-supported accounting information. This positioning differentiates GAISC from general AI literacy while retaining a clear accounting/AIS task referent.

The theoretical contribution is organized through two foundations. Socio-Technical Systems Theory (STS) explains why individual, technological, and institutional elements must be considered jointly in GenAI-enabled AIS learning, while Social Cognitive Career Theory (SCCT) provides a complementary career-development logic linking learner agency, self-efficacy, contextual supports, learning experiences, and career-related outcomes. Technology readiness, TAM, self-efficacy research, and UTAUT facilitating-condition logic are therefore used as construct-level underpinning mechanisms rather than as coequal theories. This architecture reduces conceptual inflation while preserving the rationale for all five antecedents.

Further contribution is substantive and inferential. The study examines how GAISC relates to AI ethics awareness and career readiness, and it uses endogeneity diagnostics to distinguish baseline statistical significance from relationships that remain robust after adjustment. This robustness layer is not presented as a new method; rather, it strengthens inference by showing that the relationships between facilitating conditions and GAISC and between AIEA and CR require more cautious interpretation than the other structural relationships.

## 2. Literature review, theoretical framework, hypothesis development, and research model

### Literature review

GenAI has become increasingly relevant to accounting education as accounting work becomes more data-intensive, system-mediated, and dependent on the evaluation of digital evidence. Accounting students increasingly use AI-supported tools for explanation, analytics, and help-seeking, while educators face the challenge of preserving critical thinking, verification, and professional judgment [2,3,21,22]. Pedagogical designs that require students to reperform calculations and critically review AI-generated errors illustrate why competence in accounting contexts must include verification and judgment, not only fluent interaction with AI [8]. Evidence from auditing and digital-reporting research further indicates that perceived usefulness, ease of use, facilitating conditions, organizational support, and institutional arrangements can influence how accounting technologies are adopted and embedded in practice [23–25].

Recent AI-literacy research reinforces this multidimensional view. Studies of prospective teachers and EFL educators conceptualize AI or GenAI literacy in terms that include understanding, use, evaluation, problem solving, and ethics [9,10]. These studies are informative but are not substitutes for an AIS-specific competence construct. GAISC is narrower in domain and more explicitly tied to accounting-information processing, output verification, inconsistency detection, and professional judgment. Recent evidence from Vietnamese accounting students also shows that adoption-related beliefs and facilitating conditions are salient predictors of AI adoption, making it theoretically useful to examine whether similar antecedents are associated with the more demanding outcome of AIS competence [11].

The learning environment also matters, but the presence of technology or policy is not equivalent to effective pedagogical support. Research on emerging learning spaces emphasizes that technical infrastructure, instructional design, and ethical safeguards jointly shape technology-enabled learning [26]. Likewise, evidence on AI ethics in vocational education shows that institutional policy support and individual ethical orientation can play distinct roles, cautioning against treating formal support as an automatic determinant of responsible practice [27]. These findings support the distinction between perceived facilitating conditions as a contextual enabler and learner-level agency or technology appraisal as more proximal correlates of perceived competence.

Downstream, the model connects GAISC with AI ethics awareness and career readiness. Accounting-specific GenAI use makes questions of accuracy, bias, privacy, explainability, accountability, and human oversight salient because AI-supported outputs must be evaluated against professional evidence and judgment standards [28–30]. Information-integrity research further underscores the importance of distinguishing reliable from misleading sources when users evaluate digital information [31]. At the career level, recent evidence among accounting students shows that AI-related mindset and experiential learning are associated with perceived readiness for digitally transformed accounting work [32]. Recent field evidence in auditing also identifies explainability, bias, privacy, reliability, overreliance, and governance as material AI-related concerns [33–37].

### Theoretical framework

#### Socio-Technical Systems Theory (STS).

Socio-Technical Systems Theory (STS) is the first foundational theory of the model. STS treats organizational and work outcomes as products of the joint configuration of social and technical subsystems rather than of technology in isolation [38]. In information-systems research, the socio-technical perspective similarly emphasizes the interdependence of people, technologies, tasks, and organizational arrangements [39]. Applied to GenAI-enabled AIS learning, this logic explains why perceived competence should be examined in relation to learner preparedness and agency, technology-related appraisals, and institutional/learning-environment support. The AIS context is especially suitable for an STS lens because accounting information work combines digital systems with control procedures, evidence evaluation, documentation, and human professional judgment.

#### Social Cognitive Career Theory (SCCT).

Social Cognitive Career Theory (SCCT) is the second foundational theory. SCCT explains how self-efficacy, outcome expectations, learning experiences, and contextual supports or barriers shape career-relevant interests, choices, persistence, and performance-related development [40]. The present study does not claim that every construct is a direct operationalization of a canonical SCCT variable. Rather, SCCT supplies the higher-level career-development logic for linking learner agency and contextual support to competence-related self-assessments and career readiness. DLSE is most directly aligned with the self-efficacy mechanism, FC represents perceived contextual support, and the broader set of GenAI learning experiences and appraisals provides the environment in which students form perceived competence and career-readiness judgments.

#### Organizing logic and underpinning mechanisms.

Together, STS and SCCT organize the model into three antecedent domains: individual learner preparedness and agency (GAR and DLSE), technology-related appraisals (PEOU and PU), and institutional/learning-environment support (FC). Construct-level mechanisms then provide more specific explanations: technology readiness underpins GAR; TAM underpins PEOU and PU; social-cognitive self-efficacy research clarifies DLSE; and UTAUT facilitating-condition logic clarifies FC. GAISC is the focal accounting-specific construct, while AIEA and CR are downstream outcomes supported by responsible-AI/professional-ethics and work-readiness literature. This hierarchy uses additional theories as underpinning mechanisms rather than as coequal foundations.

### Hypothesis development

#### GenAI readiness and GenAI-enabled AIS competence.

Within the STS domain of individual preparedness, GenAI readiness captures students’ cognitive and adaptive preparedness to engage with an emerging technology. Technology-readiness research suggests that preparedness influences willingness to experiment with new technologies and tolerate uncertainty [41,42]. In an AIS setting, such preparedness may be associated with greater willingness to explore GenAI-supported accounting representations, compare outputs with accounting evidence, and persist when outputs are uncertain. Recent Vietnamese evidence also links technology readiness with AI adoption among accounting students, although adoption remains conceptually distinct from AIS competence [11].

Therefore, the hypothesis is proposed: *Hypothesis 1 (H1): GAR is positively associated with GAISC.*

#### Perceived ease of use and GenAI-enabled AIS competence.

Within the STS technology-appraisal domain, perceived ease of use (PEOU) captures the effort students expect when interacting with GenAI for AIS-related learning. TAM defines PEOU as the belief that using a technology requires relatively little effort [43,44]. In the present model, TAM is an underpinning mechanism rather than a foundational theory: lower interaction effort may leave more cognitive capacity for prompt refinement, transaction interpretation, output comparison, and accounting reasoning. This competence-oriented extension is consistent with task–technology fit logic and with recent evidence that students perceive AI-mediated instruction as relevant to engagement in technology-enabled learning environments [45,46].

*Therefore, we propose the following hypothesis*

*Hypothesis 2 (H2): PEOU is positively associated with GAISC*

#### Digital learning self-efficacy and GenAI-enabled AIS competence.

SCCT gives self-efficacy a central role in learner agency and persistence [40]. Digital learning self-efficacy (DLSE) therefore captures confidence in learning, practicing, and solving problems in digital environments. Its construct-level underpinning also draws on computer and digital self-efficacy research, which links efficacy beliefs with persistence, self-regulation, feedback seeking, and continued effort when technology-mediated tasks are difficult [47–49]. In GenAI-enabled AIS learning, such agency should be positively associated with students’ perceived ability to verify and refine accounting-related AI outputs.

*Hypothesis 3 (H3): DLSE is positively associated with GAISC*

#### Facilitating conditions and GenAI-enabled AIS competence.

Within STS, facilitating conditions represent the institutional and learning-environment domain. UTAUT provides the construct-level logic that users are better positioned to engage with technology when resources, guidance, and support are available [50,51]. In GenAI-enabled AIS learning, FC includes perceived access to digital infrastructure, lecturer guidance, accounting-specific practice opportunities, feedback, and responsible-use rules. Recent research emphasizes that effective AI-enabled learning spaces depend not only on technical availability but also on pedagogical and ethical design [26], while institutional policy support may not operate uniformly across settings [27]. Thus, FC is theorized as an enabling context rather than as a guaranteed strong independent determinant.

Thus, the hypothesis is proposed

*Hypothesis 4 (H4): Facilitating conditions (FC) are positively associated with GAISC*

#### Perceived usefulness and GenAI-enabled AIS competence.

Perceived usefulness (PU) is the second technology-appraisal construct. TAM defines PU as the belief that technology can enhance relevant task performance [43,44]. In an AIS learning context, usefulness is expected to become competence-relevant when students perceive GenAI as valuable for accounting-information search, transaction interpretation, output comparison, exception checking, and evidence evaluation. Accounting-education research increasingly shows that the value of GenAI depends on purposeful task integration and critical evaluation rather than frequency of access alone [2,8]. Students who perceive stronger task value are therefore expected to report higher GAISC scores.

*Hypothesis 5 (H5): PU is positively associated with GAISC*

#### GenAI-enabled AIS competence and AI ethics awareness.

GAISC represents students’ self-assessment of their ability to use GenAI in accounting-information tasks while checking outputs, detecting inconsistencies, evaluating evidence, and exercising professional judgment. These activities expose users to ethical concerns involving accuracy, bias, privacy, explainability, accountability, and appropriate human oversight. Recent AI-literacy research explicitly treats ethics and evaluation as components of competent AI use [10], while accounting research shows that GenAI advice can influence professional and ethical judgment [30]. Algorithmic-bias research also illustrates how system design can amplify socially patterned distortions [29]. Higher GAISC scores are therefore expected to be positively associated with AIEA.

*Hypothesis 6 (H6): GAISC is positively associated with AIEA*

#### GenAI-enabled AIS competence and career readiness.

Contemporary accounting work increasingly requires graduates to combine accounting knowledge with digital systems, analytics, and judgment under technology-enabled conditions. SCCT provides the career-development logic for expecting learning experiences and competence self-assessments to be associated with career-related self-assessments [40]. In this study, CR is measured as students’ self-reported career readiness rather than verified employability or workplace performance. Students reporting stronger GAISC may also feel better prepared to evaluate AI-supported accounting outputs and operate in digitally transformed accounting environments. Recent evidence among accounting students likewise links AI-related mindset and experiential learning with career-readiness perceptions [32]. This interpretation is also consistent with employability research that emphasizes adaptability and work-relevant human capital, as well as recent reviews of accounting employability and employer demand for digital capabilities [52–58].

*Hypothesis 7 (H7): GAISC is positively associated with CR*

#### AI ethics awareness and career readiness.

AI ethics awareness reflects students’ recognition of ethical principles and risks associated with AI use, including accuracy, bias, transparency, privacy, accountability, and responsible human oversight. In accounting and auditing, these concerns are closely tied to evidence evaluation, confidentiality, documentation, and professional judgment [28,30]. Ethical awareness may complement students’ perceived preparation for professional environments in which AI-supported judgments must be checked and justified, although awareness alone is not equivalent to task competence or employability. The hypothesis therefore concerns a positive association with CR, not a claim that ethics awareness independently produces actual workplace readiness.

*Hypothesis 8 (H8): AIEA is positively associated with CR*

#### Perceived usefulness and career readiness.

PU may also be directly associated with CR because students who see GenAI as useful for authentic AIS tasks may perceive stronger alignment between their learning activities and future accounting work. This pathway is consistent with SCCT’s broader emphasis on career-relevant expectations and learning experiences, while TAM supplies the construct-level definition of usefulness [40,43]. Recent accounting evidence shows that perceived usefulness and related adoption beliefs are salient among Vietnamese accounting students [11], and research on Accountant 5.0 similarly links AI-oriented learning and career-readiness perceptions [32].

*Hypothesis 9 (H9): PU is positively associated with CR*

### Research model

The research model in Figure 1 is anchored in STS and SCCT. STS organizes the antecedents into individual learner preparedness and agency (GAR, DLSE), technology-related appraisals (PEOU, PU), and institutional/learning-environment support (FC). SCCT provides complementary logic for learner agency, contextual support, learning experiences, and career-related outcomes. Technology readiness, TAM, self-efficacy research, and UTAUT facilitating-condition logic are used only as construct-level underpinning mechanisms. GAISC is positioned as the focal accounting-specific construct, with AIEA and CR as downstream outcomes; PU and AIEA are additionally specified as direct antecedents of CR. GAISC and CR are operationalized through self-report items and are therefore interpreted as students’ assessments rather than objectively demonstrated competence or employability.

**Fig 1:**
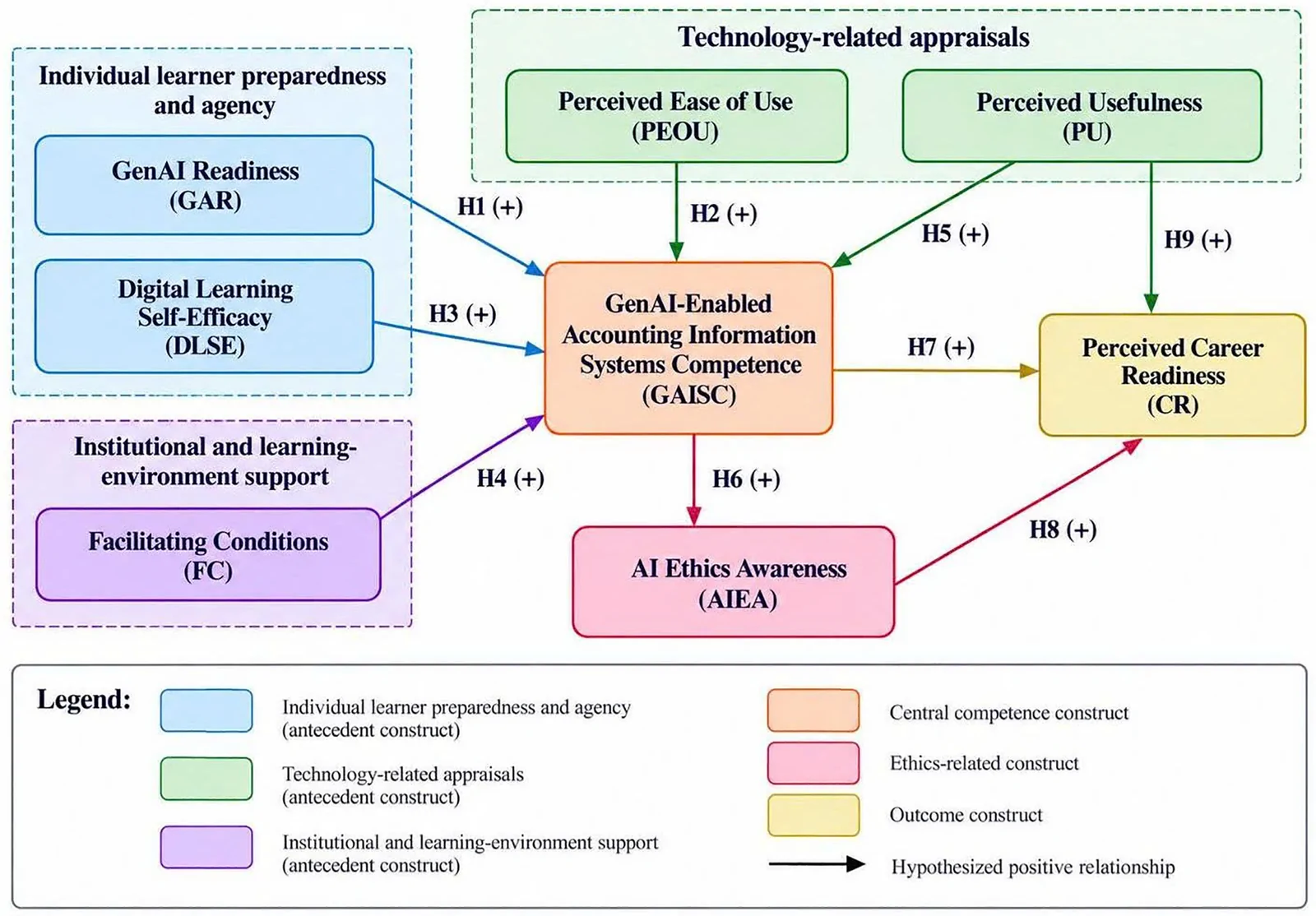
Research model.

Fig 1 presents the proposed research model. H1–H5 specify positive associations of GAR, PEOU, DLSE, FC, and PU with GAISC. H6 and H7 link GAISC with AIEA and CR, respectively. H8 and H9 specify positive associations of AIEA and PU with CR. The hypotheses are association-based because the empirical design is cross-sectional and relies on self-reported measures.

## 3. Methodology

### Research design

This study used a theory-driven, quantitative, cross-sectional survey design to examine associations among GAR, PEOU, DLSE, FC, PU, GAISC, AIEA, and CR. The model was anchored in STS and SCCT, with construct-level mechanisms drawn from technology readiness, TAM, self-efficacy research, and UTAUT facilitating-condition logic. Because all focal variables were measured at one point in time using student self-reports, the estimated structural paths were interpreted as theoretically guided associations rather than definitive causal effects [59,60].

The survey design was appropriate because the focal constructs concern students’ perceptions, learning beliefs, perceived support, competence self-assessments, ethical awareness, and career-readiness evaluations. The study therefore does not demonstrate objective AIS task performance, actual workplace behavior, or employer-verified readiness. Instead, it captures perceived competence and career-readiness judgments among students with relevant accounting, AIS, and GenAI learning exposure.

### Empirical setting, target population, and respondent logic

The empirical setting is undergraduate accounting and auditing education in the context of GenAI-enabled accounting information systems learning. This setting is appropriate because accounting students are increasingly exposed to digital accounting tools, AIS platforms, data analytics, and GenAI-supported learning activities, while the accounting profession increasingly expects graduates to combine accounting knowledge with digital competence, ethical judgment, and technology-enabled problem solving.

The target population consists of undergraduate students majoring in accounting or auditing. Eligible respondents are students who have completed foundational accounting courses and have prior exposure to AIS, digital accounting tools, or GenAI-supported learning activities. This screening criterion is necessary because the research model requires respondents to evaluate GenAI-enabled AIS competence, perceived usefulness, AI ethics awareness, and career readiness on the basis of relevant learning experience rather than on general familiarity with AI tools.

The respondent logic is student-based rather than key-informant based. Respondents are expected to provide self-assessments of their own readiness, ease-of-use perceptions, digital learning self-efficacy, perceived facilitating conditions, competence, usefulness beliefs, ethical awareness, and career readiness. Students without sufficient exposure to accounting, AIS-related learning, or GenAI-supported learning activities should be excluded because their responses may not reflect the empirical setting of the study.

### Sampling process, data collection, and sample-size justification

The study used purposive sampling combined with convenience sampling because respondents needed sufficient accounting and GenAI-related learning exposure to evaluate the focal constructs. Data were collected from 21 October to 31 December 2025 among third- and fourth-year undergraduate accounting and auditing students at five universities in Vietnam. Eligibility required completion of foundational accounting courses and prior exposure to AIS, digital accounting tools, or GenAI-supported learning activities.

Recruitment used a two-stage eligibility procedure. Lecturers assisting recruitment first conducted a preliminary check of the eligibility criteria before inviting students to participate. Students who passed this preliminary screening received an oral explanation of the study purpose, voluntary nature of participation, anonymity, the right to decline or stop without academic penalty, and the absence of rewards or bonus marks. Only students who verbally agreed to participate were handed the paper-based questionnaire. The recruiting lecturer directly heard and confirmed the oral agreement before questionnaire distribution. No names, student identification numbers, email addresses, phone numbers, or other personally identifiable information were collected.

Before the main survey, the instrument was pilot-tested with 30 eligible accounting and auditing students; pilot responses were excluded from the analytical dataset. A total of 500 students who had been pre-screened as meeting the eligibility criteria entered the questionnaire stage. During post-collection screening, eligibility was verified again using the information supplied in the questionnaire. This secondary verification identified eight cases that did not fully satisfy the final eligibility criteria. Together with 10 incomplete questionnaires, four duplicate responses, and seven unreliable response patterns, 29 questionnaires were excluded, leaving 471 valid responses. Accordingly, 471/500 = 94.2% is reported as the usable questionnaire rate rather than as a conventional survey response rate. The sampling design, respondent eligibility criteria, data-collection procedure, screening outcomes, and sample-size justification are summarized in Table 1. Because university identifiers were not recorded on individual questionnaires and completed paper questionnaires from the five sites were pooled after collection to preserve anonymity, university-specific respondent counts cannot be reconstructed retrospectively.

**Table 1:** Sampling, data collection, and sample-size parameters.

Parameter	Specification
Sampling approach	Purposive sampling combined with convenience sampling
Sampling setting	Five participating universities in Vietnam
Target respondents	Third- and fourth-year undergraduate accounting and auditing students in the participating universities
Eligibility criteria	Completion of foundational accounting courses and prior exposure to AIS, digital accounting tools, or GenAI-supported learning activities
Data collection mode	Paper-based questionnaire distributed after lecturer-assisted preliminary screening and verbal consent
Pilot test	30 students; pilot responses excluded from the final dataset
Statistical test	F-test in linear multiple regression; fixed model; R-square deviation from zero
Effect size	Medium effect size, f^2^ = .15
Significance level and power	α = .05; statistical power = .95
Number of predictors	Five predictors for GAISC: GAR, PEOU, DLSE, FC, and PU
Minimum required sample size	138 observations
10-times rule minimum	50 observations
Pre-screened students entering questionnaire stage	500
Responses excluded	29 (10 incomplete, 8 failing secondary eligibility verification, 4 duplicates, and 7 unreliable response patterns)
Valid responses retained	471
Usable questionnaire rate	94.2% (471/500)

Note: The G*Power 3.1 calculation was based on the most complex endogenous construct in the structural model. The final analytical sample of 471 exceeded both a priori G*Power requirement (138) and the supplementary ten-times-rule minimum (50). The 94.2% figure is the usable questionnaire rate calculated as 471 retained questionnaires divided by 500 students entering the questionnaire stage after preliminary eligibility screening. The a priori G*Power specification used a medium f^*²*^ *= .15 benchmark and followed established statistical-power guidance for regression-based power analysis* [61,62].

### Ethics approval and informed consent

Ethical approval was granted by University of Finance – Marketing on 20 October 2025, before data collection began. The approved protocol explicitly permitted verbal informed consent for this anonymous, minimal-risk student survey. Before a paper questionnaire was handed to a student, the lecturer assisting recruitment explained the study and asked whether the student agreed to participate. The lecturer directly heard and confirmed the student’s oral agreement; only students who agreed were given the questionnaire. A separate signed consent form was not collected, thereby preserving the anonymous survey procedure. Students were informed that participation was voluntary, that they could decline or stop at any time without academic disadvantage, and that responses would be used only for research and reported in aggregate form.

### Measurement variables

The questionnaire measured eight reflective latent constructs with four items per construct (32 indicators) on a five-point Likert scale from 1 = strongly disagree to 5 = strongly agree. Items were adapted or developed from established literature and contextualized to GenAI-enabled AIS learning. Because GAISC and CR were self-assessed, they are interpreted as self-reported constructs rather than objective task performance, verified employability, or workplace performance. The complete item wording and theoretical source mapping are provided in S1 File Supporting information.

### Data screening and descriptive analysis

Before model estimation, questionnaires were screened for completeness, secondary eligibility verification, duplicate participation, straight-lining, and consistency with the target population. Incomplete, ineligible, duplicate, or unreliable responses were removed before analysis. Screening focused on response quality and suitability for the study population; no objective task-performance or institutional-record data were used.

Descriptive statistics were calculated for the measurement indicators and latent constructs using means and standard deviations. These statistics describe perceived survey-score patterns rather than objective AIS performance, actual GenAI usage, or workplace outcomes.

### Common method bias diagnostics

Because the focal variables were collected from the same respondents using one cross-sectional questionnaire, common method bias (CMB) was addressed through procedural remedies and a common latent factor (CLF) diagnostic. Procedurally, respondents were assured of anonymity and confidentiality, told that there were no right or wrong answers, and asked to respond based on their own AIS learning experience. The questionnaire used neutral wording and separated predictor and outcome blocks to reduce evaluation apprehension and consistency-motif concerns [63].

The CLF diagnostic was estimated in AMOS/CFA by comparing the baseline measurement model with an alternative model in which observed indicators also loaded on a common latent factor. The diagnostic was used to assess whether adding a common method factor materially improved model fit; it was not used to claim that common method variance was absent. Because the data remained single-source and cross-sectional, residual common method variance could not be ruled out.

### PLS-SEM estimation strategy

The main analysis was conducted in SmartPLS using a two-stage PLS-SEM procedure. First, the reflective measurement model was assessed through outer loadings, Cronbach’s alpha, Dijkstra–Henseler’s ρA, average variance extracted (AVE), and the heterotrait-monotrait ratio (HTMT). Outer loadings of approximately .70 or higher, AVE above .50, and HTMT below the conservative .85 criterion were used as reference points for measurement quality [60,64].

After measurement quality was established, the structural model was assessed. Inner VIF values were used only to diagnose predictor collinearity. Direct relationships were evaluated using bootstrapping with 5,000 resamples. Standardized path coefficients, t-values, p-values, local f^2^ effect sizes, R^2^ adjusted R^2^, and blindfolding Q^2^ were reported. Statistical significance was interpreted together with coefficient magnitude, local effect size, and endogeneity robustness rather than as the sole indicator of substantive importance.

The structural model specified nine direct relationships: between GAR and GAISC, PEOU and GAISC, DLSE and GAISC, FC and GAISC, PU and GAISC, GAISC and AIEA, GAISC and CR, AIEA and CR, and PU and CR. Indirect effects were examined only as supplementary baseline mechanism evidence because the study was not designed to establish causal mediation.

### Predictive relevance and endogeneity diagnostics

Predictive relevance was assessed with the SmartPLS blindfolding procedure using construct cross-validated redundancy. Q^2^ values above zero were interpreted as evidence of in-sample predictive relevance for endogenous constructs, not as evidence of out-of-sample predictive performance [65].

Potential endogeneity was assessed using the Gaussian copula approach as a robustness diagnostic [19,20]. An initial full model added copula terms to the structural relationships for diagnostic purposes. A reduced model was then re-estimated after retaining the copula terms that were statistically significant at the 5% level. The substantive interpretation focused on whether each hypothesized relationship remained statistically significant and directionally stable after this model-level adjustment; non-robustness of a path was not interpreted as proof that the predictor itself was endogenous.

## 4. Results

### Common method bias diagnostic

The CLF comparison was conducted before interpreting the measurement and structural models. As shown in Table 2, adding the common latent factor did not significantly improve model fit.

**Table 2:** Common method bias diagnostics using the common latent factor approach.

Model	CMIN/χ²	df	p-value	χ²/df	Δχ²	Δdf	Conclusion
Without CLF	428.580	436	.591	.983	–	–	Baseline measurement model
With CLF	426.682	435	.603	.981	1.898	1	No significant fit improvement after adding CLF

The baseline CFA model without the CLF showed χ^2^ = 428.580, df = 436, p = .591, and χ^2^/df = .983. The model with the CLF showed χ^2^ = 426.682, df = 435, p = .603, and χ^2^/df = .981. The difference was not statistically significant, Δχ^2^(1) = 1.898, p ≈ .168. Thus, the diagnostic did not provide statistical evidence that a common method factor materially improved the measurement model or dominated the observed covariance structure. Nevertheless, because the substantive variables came from the same respondents and questionnaire at one time point, residual common method variance cannot be completely ruled out.

### Measurement model

The reflective measurement model was assessed before interpretation of the structural relationships. Table 3 reports indicator reliability, internal consistency, and convergent validity.

**Table 3:** Measurement model assessment: outer loadings, reliability, convergent validity.

Construct	Item	Outer loading	Cronbach’s alpha	Reliability (ρA)	AVE
AIEA	AIEA1	.839	.845	.846	.683
	AIEA2	.817			
	AIEA3	.833			
	AIEA4	.815			
CR	CR1	.844	.843	.846	.680
	CR2	.824			
	CR3	.808			
	CR4	.822			
DLSE	DLSE1	.843	.841	.851	.676
	DLSE2	.837			
	DLSE3	.809			
	DLSE4	.798			
FC	FC1	.833	.854	.866	.694
	FC2	.815			
	FC3	.832			
	FC4	.853			
GAISC	GAISC1	.868	.874	.875	.726
	GAISC2	.843			
	GAISC3	.844			
	GAISC4	.853			
GAR	GAR1	.834	.855	.858	.696
	GAR2	.829			
	GAR3	.845			
	GAR4	.831			
PEOU	PEOU1	.831	.860	.861	.704
	PEOU2	.831			
	PEOU3	.852			
	PEOU4	.843			
PU	PU1	.851	.850	.853	.689
	PU2	.848			
	PU3	.804			
	PU4	.816			

All outer loadings ranged from .798 to .868. Cronbach’s alpha values ranged from .841 to .874, ρA values from .846 to .875, and AVE values from .676 to .726. These results support indicator reliability, internal consistency, and convergent validity for the eight reflective constructs.

All HTMT values in Table 4 were below .85, supporting empirical distinctiveness among the constructs. The comparatively higher HTMT values among PU, GAISC, AIEA, and CR are theoretically plausible because these constructs occupy adjacent appraisal, competence, and outcome positions in the model, but none exceeded the prespecified criterion.

**Table 4:** Discriminant Validity Assessment Using the HTMT Criterion.

	AIEA	CR	DLSE	FC	GAISC	GAR	PEOU	PU
AIEA								
CR	.731							
DLSE	.437	.400						
FC	.377	.346	.064					
GAISC	.785	.734	.407	.347				
GAR	.408	.408	.180	.133	.426			
PEOU	.405	.430	.123	.143	.489	.093		
PU	.767	.778	.371	.398	.762	.431	.410	

### Structural model: collinearity, effect sizes, and baseline direct relationships

Table 5 reports inner VIF values and local f^2^ effect sizes. VIF values ranged from 1.000 to 2.171, indicating no serious predictor-collinearity concern. The VIF results are interpreted solely as structural-collinearity diagnostics and not as a test of common method bias.

**Table 5:** Inner VIF and f^2.^

Path	VIF	f^2^	Effect size
AIEA - > CR	2.130	.048	Small
DLSE - > GAISC	1.136	.056	Small
FC - > GAISC	1.158	.023	Small
GAISC - > AIEA	1.000	.838	Large
GAISC - > CR	2.171	.062	Small
GAR - > GAISC	1.164	.044	Small
PEOU - > GAISC	1.144	.098	Small
PU - > CR	2.046	.128	Small
PU - > GAISC	1.596	.244	Medium

The local effect-size pattern shows a clear substantive hierarchy. GAISC - > AIEA had a large f^2^ of .838, while PU - > GAISC had a medium f^2^ of .244 according to conventional benchmarks. The remaining local f^2^ values were smaller: PU - > CR = .128, PEOU - > GAISC = .098, GAISC - > CR = .062, DLSE - > GAISC = .056, AIEA - > CR = .048, GAR - > GAISC = .044, and FC - > GAISC = .023. These benchmarks are used as heuristics; a small f^2^ is not equated with practical irrelevance.

Fig 2 presents the baseline structural model. All nine hypothesized paths were statistically significant before endogeneity adjustment. Within the GAISC equation, PU showed the largest standardized coefficient (β = .427), followed by PEOU (.229), DLSE (.172), GAR (.154), and FC (.113). GAISC was strongly associated with AIEA (β = .675) and positively associated with CR (β = .252); PU (β = .352) and AIEA (β = .220) were also positively associated with CR in the baseline model.

**Fig 2:**
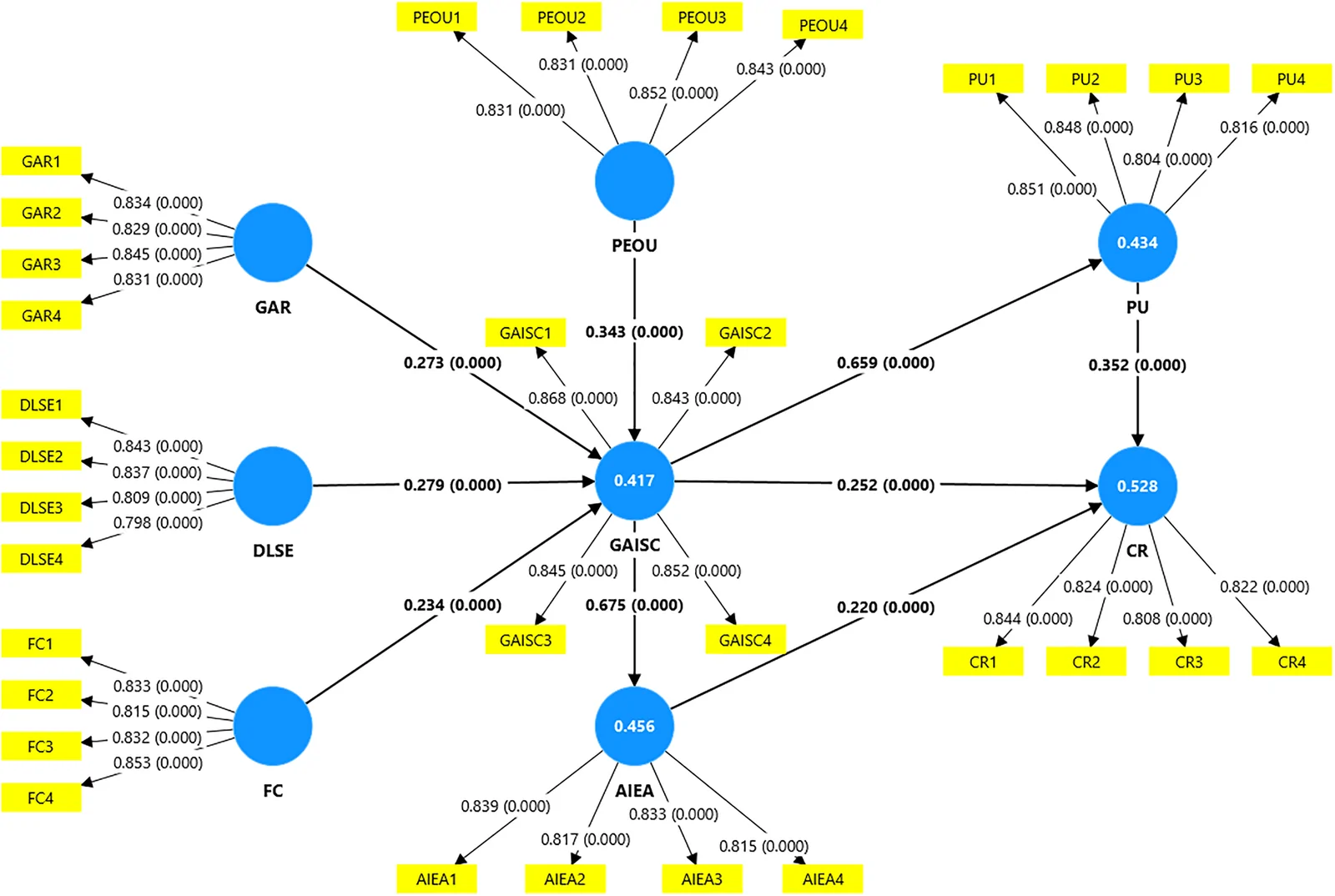
Baseline bootstrapped PLS-SEM results of the research model.

### Explanatory power and predictive relevance

Table 6 shows that the model explained 45.6% of the variance in AIEA, 52.9% in CR, and 53.1% in GAISC. These R^2^ values describe the explanatory capacity of each full structural equation and are not interpreted as the importance of any single predictor.

**Table 6:** R^2^ and adjusted R^2.^

	R^2^	Adjusted R^2^
AIEA	.456	.455
CR	.529	.526
GAISC	.531	.526

As shown in Table 7, blindfolding produced Q^2^ values above zero for GAISC (.379), CR (.352), and AIEA (.308), indicating in-sample predictive relevance. These values are not interpreted as out-of-sample predictive performance.

**Table 7:** In-sample predictive relevance using blindfolding.

Endogenous construct	SSO	SSE	Q^2^ (= 1 − SSE/SSO)	Interpretation
GAISC	1884.000	1169.913	.379	In-sample predictive relevance established
CR	1884.000	1220.716	.352	In-sample predictive relevance established
AIEA	1884.000	1303.699	.308	In-sample predictive relevance established

### Endogeneity and robustness

Gaussian copula diagnostics were estimated in two stages. In the initial full model, significant copula terms at the 5% level were observed for FC - > GAISC, GAISC - > AIEA, GAISC - > CR, PU - > CR, and PU - > GAISC; AIEA - > CR had a marginal copula term (p = .059) and was not retained under the 5% decision rule. The reduced model retained only the statistically significant copula terms. Seven hypothesized relationships remained positive and statistically significant after adjustment: GAR - > GAISC (β = .194, p < .001), PEOU - > GAISC (β = .271, p < .001), DLSE - > GAISC (β = .191, p < .001), PU - > GAISC (β = .332, p < .001), GAISC - > AIEA (β = .858, p < .001), GAISC - > CR (β = .373, p < .001), and PU - > CR (β = .420, p < .001). By contrast, FC - > GAISC became non-significant (β = −.019, p = .811), and AIEA - > CR also became non-significant (β = .103, p = .085). Thus, H4 and H8 are baseline-supported but not robust after endogeneity adjustment. The full and reduced copula outputs are reported in S1 File Supporting information, and the reduced model is visualized in S1 File Supporting information.

Table 8 makes the baseline-versus-robustness distinction explicit. All nine hypotheses received baseline statistical support, but only seven relationships remained robust after adjustment. The largest baseline antecedent of GAISC was PU, whereas FC was the smallest and also non-robust. GAISC - > AIEA was substantively strong and remained robust. For CR, PU and GAISC retained robust direct associations, whereas the smaller baseline AIEA - > CR relationship did not.

**Table 8:** Baseline and endogeneity-adjusted structural relationships.

Hypothesis	Path	Baseline β	Baseline p	f^2^	Adjusted β	Adjusted p	Robustness interpretation
H1	GAR - > GAISC	.154	< .001	.044	.194	< .001	Baseline-supported; robust after adjustment
H2	PEOU - > GAISC	.229	< .001	.098	.271	< .001	Baseline-supported; robust after adjustment
H3	DLSE - > GAISC	.172	< .001	.056	.191	< .001	Baseline-supported; robust after adjustment
H4	FC - > GAISC	.113	.002	.023	−.019	.811	Baseline-supported; not robust after adjustment
H5	PU - > GAISC	.427	< .001	.244	.332	< .001	Baseline-supported; robust after adjustment
H6	GAISC - > AIEA	.675	< .001	.838	.858	< .001	Baseline-supported; robust after adjustment
H7	GAISC - > CR	.252	< .001	.062	.373	< .001	Baseline-supported; robust after adjustment
H8	AIEA - > CR	.220	< .001	.048	.103	.085	Baseline-supported; not robust after adjustment
H9	PU - > CR	.352	< .001	.128	.420	< .001	Baseline-supported; robust after adjustment

*Note: Baseline β and p values are from the bootstrapped PLS-SEM model; f² values are baseline local effect sizes. Adjusted β and p values are from the reduced Gaussian copula model after retaining the copula terms significant at the 5% level. “Not robust” means that the baseline association did not remain statistically significant after the model-level endogeneity adjustment; it does not imply that the construct is unimportant or that the predictor itself has been proven endogenous.*

### Supplementary indirect effects

The supplementary indirect effects are consistent with GAISC acting as an important intermediate construct in the baseline model. However, because the study is cross-sectional and the endogeneity analysis did not re-estimate every indirect pathway as a causal mediation model, these results are interpreted as descriptive baseline mechanism evidence. Indirect pathways involving FC - > GAISC or AIEA - > CR receive additional caution because one of their component direct relationships was non-robust after adjustment (Table 9).

**Table 9:** Supplementary baseline PLS-SEM specific indirect effects.

Indirect path	Original sample (O)	Sample mean (M)	STDEV	t-value	p-value	Baseline decision / robustness interpretation
GAR - > GAISC - > CR	.039	.040	.014	2.833	.005	Baseline supported
PU - > GAISC - > CR	.108	.109	.028	3.822	< .001	Baseline supported
GAR - > GAISC - > AIEA	.104	.105	.024	4.297	< .001	Baseline supported
GAISC - > AIEA - > CR	.149	.148	.034	4.357	< .001	Baseline supported; interpreted cautiously because AIEA - > CR is not robust after copula adjustment
PEOU - > GAISC - > CR	.058	.059	.017	3.315	.001	Baseline supported
PEOU - > GAISC - > AIEA - > CR	.034	.034	.009	3.896	< .001	Baseline supported; interpreted cautiously because AIEA - > CR is not robust after copula adjustment
PU - > GAISC - > AIEA	.289	.288	.035	8.239	< .001	Baseline supported
DLSE - > GAISC - > AIEA - > CR	.026	.026	.008	3.369	.001	Baseline supported; interpreted cautiously because AIEA - > CR is not robust after copula adjustment
FC - > GAISC - > AIEA	.076	.078	.026	2.977	.003	Baseline supported; interpreted cautiously because FC - > GAISC is not robust after copula adjustment
DLSE - > GAISC - > CR	.043	.045	.014	3.071	.002	Baseline supported
DLSE - > GAISC - > AIEA	.116	.118	.023	5.100	< .001	Baseline supported
FC - > GAISC - > AIEA - > CR	.017	.017	.007	2.513	.012	Baseline supported; interpreted cautiously because both FC - > GAISC and AIEA - > CR are not robust after copula adjustment
PEOU - > GAISC - > AIEA	.155	.156	.025	6.278	< .001	Baseline supported
FC - > GAISC - > CR	.028	.030	.012	2.294	.022	Baseline supported; interpreted cautiously because FC - > GAISC is not robust after copula adjustment
PU - > GAISC - > AIEA - > CR	.064	.063	.016	3.856	< .001	Baseline supported; interpreted cautiously because AIEA - > CR is not robust after copula adjustment
GAR - > GAISC - > AIEA - > CR	.023	.023	.007	3.177	.001	Baseline supported; interpreted cautiously because AIEA - > CR is not robust after copula adjustment

*Note:* Table 9 *reports supplementary baseline PLS-SEM indirect effects before Gaussian copula adjustment. All indirect effects were statistically significant in the baseline model. Paths containing FC - > GAISC and/or AIEA - > CR should be interpreted cautiously because those component relationships were not robust in the reduced Gaussian copula model. The table therefore provides baseline indirect-effect evidence rather than endogeneity-robust causal mediation evidence.*

## 5. Discussion

### Perceived usefulness as the dominant antecedent of GAISC

The first headline finding is the relative importance of perceived usefulness. PU showed the largest baseline coefficient among the five antecedents of GAISC (β = .427) and the largest local contribution in that equation (f^2^ = .244); it also remained robust after endogeneity adjustment. This pattern suggests that perceived task value is more central to students’ GAISC self-assessments than ease of interaction, general readiness, self-efficacy, or the mere availability of support. In accounting education, GenAI becomes competence-relevant when students see it as useful for authentic information-processing, verification, exception-checking, and judgment tasks. This interpretation extends adoption-oriented findings among Vietnamese accounting students [11] toward an accounting-specific competence outcome and is consistent with evidence that GenAI learning benefits depend on expertise and purposeful task integration [2,8].

### GAISC and AI ethics awareness

The second headline finding is the strong and robust association between GAISC and AIEA (baseline β = .675, f^2^ = .838; adjusted β = .858, p < .001). The magnitude is theoretically plausible because the GAISC items emphasize verification, error detection, and professional judgment rather than generic tool familiarity. Students who report stronger competence in checking AI-supported accounting information are also more likely to recognize risks involving accuracy, bias, privacy, opacity, accountability, and human oversight. GenAI-literacy research similarly treats evaluation and ethics as intertwined dimensions of competent use [10], while recent accounting evidence shows that GenAI advice can shape ethical and professional judgment [30]. Algorithmic-bias research further shows that ethical risk can be embedded in system design rather than residing only in individual users [29]. The finding therefore supports a socio-technical interpretation of responsible competence, while the cross-sectional design prevents a claim that GAISC causes ethical awareness. Because GAISC is self-reported, this interpretation concerns students’ competence assessments rather than observed AIS task performance.

### Why facilitating conditions are comparatively weak and non-robust

FC was the smallest baseline antecedent of GAISC (β = .113, f^2^ = .023) and became non-significant after endogeneity adjustment (β = −.019, p = .811). This does not imply that institutional support is irrelevant. Rather, it suggests that students’ perceptions of infrastructure, guidance, practice opportunities, and responsible-use rules may capture the availability of support without fully capturing its quality, intensity, or pedagogical effectiveness. Institutional quality, lecturer capability, curriculum design, prior AI exposure, and the authenticity of AIS tasks may therefore condition the apparent FC–GAISC relationship. Research on emerging learning spaces likewise emphasizes that technical resources must be integrated with pedagogical and ethical design [26], and evidence from AI ethics in education shows that formal policy support may not operate uniformly across contexts [27]. FC is therefore better interpreted as a contextual enabling condition than as a robust independent driver of GAISC.

### Why AIEA does not retain an independent robust relationship with career readiness

AIEA was positively associated with CR in the baseline model (β = .220, f^2^ = .048), but the relationship weakened and became non-significant after adjustment (β = .103, p = .085). Ethical awareness remains professionally important, but recognizing ethical risks is not equivalent to feeling capable of performing accounting work. Career readiness may be more proximally related to task-specific competence and perceived usefulness, both of which retained robust direct associations with CR. This distinction is consistent with emerging career-readiness research among accounting students, which highlights AI-oriented learning, experiential capability, and broader work-relevant skills rather than ethical awareness alone [32]. The result therefore argues against treating ethics education as an automatic independent route to career readiness; ethics is better understood as a necessary professional dimension that complements, rather than substitutes for, competence and task value. Because CR is self-reported, this finding concerns students’ readiness assessments and should not be read as evidence of actual employability or workplace performance.

### Theoretical synthesis

Taken together, the findings support the two-theory architecture. STS explains why GAISC is situated at the intersection of learner characteristics, technology appraisals, and institutional context, but the results show that these socio-technical elements do not carry equal empirical weight. SCCT complements this account by emphasizing learner agency, self-efficacy, contextual supports, learning experiences, and career-related self-evaluations. Importantly, the study does not operationalize the full canonical SCCT model; rather, SCCT provides the career-development logic within which competence self-assessments and contextual support can be linked to career readiness. The evidence hierarchy places task value and accounting-specific GAISC at the center, with facilitating conditions and ethics awareness functioning as more contextual or complementary elements.

## 6. Theoretical and practical implications

Theoretically, the study narrows the identity of GenAI accounting-education research from generic adoption toward accounting-specific GAISC. The findings suggest that acceptance-related beliefs become more informative when they are linked to the discipline-specific ability to process, verify, and judge AI-supported accounting information. The distinction between STS/SCCT as foundations and TAM, technology readiness, self-efficacy, and UTAUT as construct-level mechanisms also provides a more parsimonious explanation of why the model contains five antecedents without treating multiple theories as coequal.

Practically, accounting educators should prioritize authentic AIS tasks in which students must verify AI-generated outputs, compare them with accounting evidence, identify inconsistencies, document reasoning, and justify professional judgments. Such designs respond to concerns about superficial or uncritical GenAI use [1] and align with pedagogical evidence favoring critical error review and reperformance [8]. GenAI-mediated learning environments should also support learner engagement and agency rather than simply provide access to tools [46].

Institutional action should focus on the quality of enabling conditions rather than the mere presence of infrastructure or policy. Clear responsible-use guidance, instructor capability, feedback routines, and task-specific practice remain important even though FC did not show a robust independent relationship with GAISC. Similarly, ethics education should be integrated with verification and professional-judgment practice because ethical awareness was strongly related to GAISC but did not independently retain a robust direct association with CR. Information-integrity research also reinforces the need to teach students how to distinguish reliable from misleading digital information rather than treating fluent AI output as inherently trustworthy [31]. These recommendations are based on self-reported GAISC and CR and should not be interpreted as evidence that the observed relationships translate directly into objective task performance or actual employability.

## 7. Limitations and future research

This study has several limitations. First, the cross-sectional, single-source, self-reported design limits strong causal and temporal inference; the structural paths therefore represent theoretically guided associations. Second, purposive and convenience sampling across five participating Vietnamese universities limits generalizability, and the sample should not be treated as nationally representative of Vietnamese accounting students. Third, GAISC and CR were measured through students’ self-assessments rather than objective AIS task performance, employer-rated readiness, actual employability, or workplace performance. Fourth, the CLF diagnostic did not indicate that a common method factor dominated the covariance structure, but residual common method variance cannot be ruled out. Fifth, the endogeneity analysis showed that FC - > GAISC and AIEA - > CR were significant in the baseline model but not robust after adjustment; related indirect paths should therefore be interpreted as baseline mechanism evidence rather than causal mediation.

Future research should combine self-reports with objective evidence such as AIS assignment performance, case-based assessments, GenAI usage logs, instructor ratings, or employer feedback. Longitudinal and experimental designs could test how accounting-specific GenAI competence develops over time and whether it predicts later workplace outcomes. Multi-institution and cross-country probability-oriented sampling would strengthen external validity. Future models should also measure instructor competence, institutional digital maturity, curriculum design, prior AI exposure, task complexity, responsible-use policy quality, and specific GenAI tools to clarify why facilitating conditions appear weak once endogeneity is addressed. Multi-source designs would further reduce common-method concerns and permit stronger tests of the relationship between ethics, competence, and career outcomes.

## 8. Conclusion

All nine hypothesized relationships were statistically significant in the baseline PLS-SEM model; seven remained robust after endogeneity adjustment, whereas FC - > GAISC and AIEA - > CR did not. This distinction is central to the final interpretation of the study. Among the five antecedents of GAISC, PU showed the strongest baseline association and the largest local f² contribution, while FC was the weakest and non-robust. GAISC showed a particularly strong and robust association with AIEA.

The substantive pattern indicates that accounting-specific task value and GAISC occupy more central positions in the model than the mere availability of facilitating conditions. For career readiness, PU and GAISC retained robust direct relationships, whereas the smaller baseline AIEA - > CR relationship did not. Ethical awareness therefore remains professionally important but should not be interpreted as an independently robust determinant of career readiness.

Overall, the study advances GenAI accounting-education research beyond generic acceptance by positioning GAISC as an accounting-specific construct connecting socio-technical learning conditions with ethical and career-related outcomes under an STS–SCCT framework. Because GAISC and CR were measured through student self-reports and the design was cross-sectional, the findings should be interpreted as association-based and endogeneity-aware evidence rather than as objective demonstrations of competence, employability, or causal effects.

## Supporting information

S1 File Supporting information. Includes S1 Table (measurement variables and theoretical sources), S2 Table (initial full Gaussian copula endogeneity diagnostics for all structural paths), S3 Table (reduced Gaussian copula model after retaining significant endogeneity terms), and S1 Figure (Gaussian copula-based endogeneity assessment in the structural model).
(DOCX)
