# Supplementary material for: GenAI-enabled accounting information systems competence: Antecedents, perceived usefulness, AI ethics awareness, and career readiness outcomes
Source: PLoS One. 2026 Nov 13;21(11):e0362651. doi: 10.1371/journal.pone.0362651 (PMC13766252; doi:10.1371/journal.pone.0362651)
Supplement: Supplementary file 1 [file pone.0362651.s001.docx]

**Supporting Information**

**GenAI-Enabled Accounting Information Systems Competence: Antecedents, Perceived Usefulness, AI Ethics Awareness, and Career Readiness Outcomes**

# S1 Table. Measurement variables and theoretical sources

| **Factor** | **Code** | **Items** | **Sources** |
| --- | --- | --- | --- |
| GenAI Readiness | GAR1 | I am prepared to use GenAI tools in accounting and AIS-related learning tasks. | Items adapted from technology readiness research [41,42] and contextualized to GenAI-enabled accounting/AIS learning based on recent GenAI/accounting research [25]. |
|  | GAR2 | I am willing to explore new GenAI functions when learning accounting information systems. |  |
|  | GAR3 | I can adapt to GenAI-supported learning activities in accounting and auditing courses. |  |
|  | GAR4 | I am ready to deal with uncertainty when using GenAI for accounting information tasks. |  |
| Perceived Ease of Use of GenAI in AIS Learning | PEOU1 | Learning to use GenAI tools for AIS-related tasks is easy for me. | Items adapted from the TAM literature [43,44] and contextualized to GenAI use in AIS learning among Vietnamese accounting and auditing students. |
|  | PEOU2 | Interacting with GenAI in AIS learning is clear and understandable. |  |
|  | PEOU3 | It is easy for me to write and revise prompts when using GenAI for AIS-related learning tasks. |  |
|  | PEOU4 | Using GenAI for AIS learning does not require excessive effort. |  |
| Digital Learning Self-Efficacy for AIS Learning | DLSE1 | I am confident that I can learn AIS concepts effectively in digital learning environments. | Items adapted from self-efficacy and computer/digital learning self-efficacy research [47,48,49] and contextualized to GenAI-supported AIS learning in Vietnamese higher education. |
|  | DLSE2 | I can complete AIS-related learning tasks using digital tools when required. |  |
|  | DLSE3 | I can solve AIS learning problems with digital tools even when the tasks are complex. |  |
|  | DLSE4 | I can improve my AIS learning by using digital feedback and correcting my mistakes. |  |
| Facilitating Conditions for GenAI-Enabled AIS Learning | FC1 | I have adequate access to digital infrastructure and GenAI tools for AIS learning. | Items adapted from UTAUT facilitating-condition research [50,51] and contextualized to infrastructure, instructor guidance, practice opportunities, and responsible GenAI-use rules in AIS learning. |
|  | FC2 | My instructors provide sufficient guidance on how to use GenAI for AIS-related tasks. |  |
|  | FC3 | My accounting/AIS courses provide practice opportunities for applying GenAI to accounting information tasks. |  |
|  | FC4 | Clear rules or guidelines are available to help students use GenAI responsibly in AIS learning. |  |
| Perceived Usefulness of GenAI in AIS Learning | PU1 | GenAI improves my ability to complete AIS-related learning tasks. | Items adapted from the TAM and task–technology fit literature [43,44,45] and contextualized to GenAI-supported AIS task performance and accounting career preparation. |
|  | PU2 | GenAI is useful for interpreting and evaluating accounting information in AIS learning. |  |
|  | PU3 | Using GenAI helps me connect AIS learning with future accounting work. |  |
|  | PU4 | GenAI supports my preparation for GenAI-enabled accounting tasks in professional contexts. |  |
| GenAI-Enabled AIS Competence | GAISC1 | I can use GenAI to process accounting information in AIS-related tasks. | Items developed based on AIS competence, accounting analytics, explainable AI, and AI-enabled accounting literature [5,12,13,36,37] and contextualized to GenAI-enabled AIS competence among Vietnamese accounting and auditing students. |
|  | GAISC2 | I can verify the accuracy of AI-generated outputs in accounting and AIS tasks. |  |
|  | GAISC3 | I can identify inconsistencies or errors in GenAI-supported accounting information. |  |
|  | GAISC4 | I can apply professional judgment when using GenAI-generated outputs in AIS-related tasks. |  |
| AI Ethics Awareness in GenAI-Enabled AIS Use | AIEA1 | I am aware that AI-generated accounting outputs may contain inaccuracies. | Items developed based on responsible AI and accounting AI ethics literature [34,35,36,37] and contextualized to responsible GenAI use in AIS-related accounting tasks. |
|  | AIEA2 | I recognize that GenAI-supported accounting information may involve bias. |  |
|  | AIEA3 | I understand privacy and confidentiality risks when using GenAI with accounting data. |  |
|  | AIEA4 | I recognize that GenAI use in AIS tasks requires transparency, explainability, accountability, and responsible human oversight. |  |
| Career Readiness for GenAI-Enabled Accounting Work | CR1 | I feel ready to perform accounting work in GenAI-enabled professional environments. | Items adapted from work readiness and employability research [53,54,55] and contextualized to GenAI-enabled accounting employability based on recent accounting education research [57,58]. |
|  | CR2 | I am confident in applying GenAI-enabled AIS competence to future accounting jobs. |  |
|  | CR3 | I am prepared to evaluate AI-supported accounting outputs in professional accounting settings. |  |
|  | CR4 | My GenAI-enabled AIS learning improves my readiness for future accounting employment. |  |

*Note: All constructs are modeled reflectively. GAISC and CR are self-reported/perceived constructs. Reference numbers in S1 Table correspond to the Vancouver-numbered reference list in the main manuscript.*

# S2 Table. Initial full Gaussian copula endogeneity diagnostics for all structural paths

| **Path / copula term** | **Original sample (O)** | **Sample mean (M)** | **STDEV** | **t** | **p** |
| --- | --- | --- | --- | --- | --- |
| AIEA -> CR | .212 | .210 | .084 | 2.506 | .012 |
| DLSE -> GAISC | .067 | .076 | .090 | .744 | .457 |
| FC -> GAISC | -.033 | -.026 | .080 | .419 | .675 |
| GAISC -> AIEA | .858 | .853 | .032 | 26.610 | < .001 |
| GAISC -> CR | .328 | .334 | .088 | 3.710 | < .001 |
| GAR -> GAISC | .085 | .085 | .133 | .641 | .522 |
| GC(AIEA -> CR) -> CR | -.087 | -.086 | .046 | 1.890 | .059 |
| GC(DLSE -> GAISC) -> GAISC | .089 | .085 | .071 | 1.253 | .210 |
| GC(FC -> GAISC) -> GAISC | .121 | .117 | .057 | 2.121 | .034 |
| GC(GAISC -> AIEA) -> AIEA | -.232 | -.225 | .047 | 4.972 | < .001 |
| GC(GAISC -> CR) -> CR | -.119 | -.120 | .038 | 3.166 | .002 |
| GC(GAR -> GAISC) -> GAISC | .066 | .068 | .127 | .518 | .604 |
| GC(PEOU -> GAISC) -> GAISC | .127 | .200 | .200 | .634 | .526 |
| GC(PU -> CR) -> CR | -.149 | -.145 | .047 | 3.148 | .002 |
| GC(PU -> GAISC) -> GAISC | .090 | .091 | .045 | 2.013 | .044 |
| PEOU -> GAISC | .103 | .033 | .204 | .507 | .612 |
| PU -> CR | .393 | .387 | .074 | 5.319 | < .001 |
| PU -> GAISC | .310 | .306 | .076 | 4.077 | < .001 |

*Note: The initial full copula model was used diagnostically. Copula terms significant at the 5% level were retained for the reduced model. The AIEA* -> *CR copula term was marginal (p = .059) and was not retained under the 5% rule.*

# S3 Table. Reduced Gaussian copula model after retaining significant endogeneity terms

| **Path / retained copula term** | **Original sample (O)** | **Sample mean (M)** | **STDEV** | **t** | **p** |
| --- | --- | --- | --- | --- | --- |
| AIEA -> CR | .103 | .102 | .060 | 1.725 | .085 |
| DLSE -> GAISC | .191 | .193 | .035 | 5.468 | < .001 |
| FC -> GAISC | -.019 | -.011 | .079 | .239 | .811 |
| GAISC -> AIEA | .858 | .853 | .032 | 26.610 | < .001 |
| GAISC -> CR | .373 | .379 | .085 | 4.408 | < .001 |
| GAR -> GAISC | .194 | .194 | .039 | 4.968 | < .001 |
| GC(FC -> GAISC) -> GAISC | .224 | .212 | .097 | 2.311 | .021 |
| GC(GAISC -> AIEA) -> AIEA | -.232 | -.225 | .047 | 4.972 | < .001 |
| GC(GAISC -> CR) -> CR | -.110 | -.110 | .037 | 3.001 | .003 |
| GC(PU -> CR) -> CR | -.135 | -.130 | .046 | 2.962 | .003 |
| GC(PU -> GAISC) -> GAISC | .175 | .167 | .072 | 2.425 | .015 |
| PEOU -> GAISC | .271 | .271 | .039 | 6.911 | < .001 |
| PU -> CR | .420 | .414 | .074 | 5.696 | < .001 |
| PU -> GAISC | .332 | .333 | .074 | 4.466 | < .001 |

*Note: Seven hypothesized relationships remain statistically significant after the retained copula terms are modeled. FC* -> *GAISC and AIEA* -> *CR are non-significant in the reduced model.*

# S1 Fig. Gaussian copula-based endogeneity assessment in the structural model

*Note: The figure visualizes the reduced Gaussian copula model and the retained copula terms. P values displayed as .000 in the SmartPLS output are reported in the manuscript as p < .001.*
